# PhenStat: A Tool Kit for Standardized Analysis of High Throughput Phenotypic Data

**DOI:** 10.1371/journal.pone.0131274

**Published:** 2015-07-06

**Authors:** Natalja Kurbatova, Jeremy C. Mason, Hugh Morgan, Terrence F. Meehan, Natasha A. Karp

**Affiliations:** 1 The EMBL—European Bioinformatics Institute, Wellcome Trust Genome Campus, Hinxton, Cambridgeshire, United Kingdom; 2 Medical Research Council Harwell, Harwell, Oxfordshire, United Kingdom; 3 Mouse Informatics Group, Wellcome Trust Sanger Institute, Hinxton, Cambridgeshire, United Kingdom; University of Westminster, UNITED KINGDOM

## Abstract

The lack of reproducibility with animal phenotyping experiments is a growing concern among the biomedical community. One contributing factor is the inadequate description of statistical analysis methods that prevents researchers from replicating results even when the original data are provided. Here we present PhenStat – a freely available R package that provides a variety of statistical methods for the identification of phenotypic associations. The methods have been developed for high throughput phenotyping pipelines implemented across various experimental designs with an emphasis on managing temporal variation. PhenStat is targeted to two user groups: small-scale users who wish to interact and test data from large resources and large-scale users who require an automated statistical analysis pipeline. The software provides guidance to the user for selecting appropriate analysis methods based on the dataset and is designed to allow for additions and modifications as needed. The package was tested on mouse and rat data and is used by the International Mouse Phenotyping Consortium (IMPC). By providing raw data and the version of PhenStat used, resources like the IMPC give users the ability to replicate and explore results within their own computing environment.

## Introduction

Irreproducibility of animal research is slowing advancement in understanding disease mechanisms, squandering resources on unproductive avenues of research and contributing to the cost of development of new drugs [1]. Funding bodies and scientific journals are addressing these concerns by forming policies that require transparent reporting of experimental design and data analysis [2]. The Animal Research: Reporting of In Vivo Experiments (ARRIVE) guidelines were published to aid authors in transparent reporting of biomedical animal studies [3]. The guidelines consist of twenty elements including clear statement of the goals of the animal study, the procedures used, the experimental design, and the analysis. The guidelines require the results of statistical analysis to be reproducible by being well documented and easily accessible.

Phenotyping, the assessment of the observable physical or biochemical characteristics of an organism, is an active area of research to understand the interaction between genotype and phenotype. A recent publication considering how to address shortcomings in phenotyping data and studies made a call for transparent reproducible phenotype associations [4]. Several high-throughput animal phenotyping projects are underway. The International Mouse Phenotyping Consortium (IMPC) aims to phenotype knockouts for all protein coding genes in the mouse genome, building on the large collection of targeted alleles in C57BL/6N embryonic stem cells available from the International Knockout Mouse Consortium [5–7]. Large-scale phenotyping is not restricted to those associated with IMPC, for example there are other institutes conducting high throughput phenotyping such as the Australian Phenomics Facility (http://www.apf.edu.au) or the Mutagenetix project at the University of Texas SouthWestern Medical Center (http://mutagenetix.utsouthwestern.edu/) and projects focused on other species for example rats (http://rgd.mcw.edu/wg/physiology), dogs (http://www.caninephenome.org), zebrafish [8] and Xenopus (http://www.sanger.ac.uk/research/projects/vertebratedevelopment/xtpp.html).

In large-scale model organism screens, a suite of statistical tests is required to accurately associate the interaction between genotype and phenotype. A phenotyping pipeline can be defined as a sequence of phenotyping procedures carried out at specific development stages or time points. Standardised protocols defining collection of data are necessary but not sufficient to identify the best statistical test to apply. The workflow–the practical implementation of a pipeline–must also be considered, as implementation varies over time and between centres. Each workflow is a balance of resources, the local specific goals, and throughput requirements. Differences in the number and frequency of controls, whether controls are measured concurrently with experimental animals, and blinding methodologies are common variables in workflows that influence which statistical test should be used. Batch in particular (defined here as those readings collected on the same day) is a significant source of variation [9] that is critical in determining how data from a pipeline should be analysed [10]. Other complicating factors include low number of animals and the influence of shared microbiome between cage mates [11]. High-throughput methods ensure large volumes of phenotype data continue to be collected, thus, an automated statistical method selection process and analysis platform is required. To promote reproducibility, the selection process and statistical methods must be available to outside researchers so results may be duplicated and further explored.

We propose that packages of tools prepared using the R environment [12] and made available via the Bioconductor package repository [13] are one of the best solutions to achieve these goals as R is widely used by the bioinformatics community and is freely available. Thus we have developed PhenStat, an R package of tools for the identification of phenotypic associations with an emphasis on statistical tools for high-throughput experiments that is made freely available from the Bioconductor repository. To encourage use and take up by the biological community, PhenStat provides an easy to use three-step process, regardless of the analysis method implemented. The first step is to perform dataset checks to ensure the analysis is appropriate and, in interactive mode, provides clear feedback about the steps taken and any issues identified. Furthermore, we have developed a function that suggests a suitable analysis method depending on the dataset characteristics. All methods output a statistical significance measure, an effect size measure, model diagnostics (when appropriate), and graphical visualisation of the genotype effect. There has been a recent call to ensure *in-vivo* experiments investigate both sexes due to the importance of understanding potential sexual dimorphic phenotypes [14]. To support this, where possible, the analysis tests whether the genotype effect has sexual dimorphism and then classifies the effect (for example: male only). Depending on the user needs, the statistical analysis output can either be interactive where the user views the graphical output and analysis summaries, or for a database implementation where the output consists of data vectors and saved graphical files.

The PhenStat package has been tested and demonstrated with an application of 420 lines of mouse phenotyping data from the http://www.sanger.ac.uk/mouseportal/ Sanger Mouse Genetics Project [15] and http://www.eumodic.org/ EUMODIC project [16] and on rat phenotyping datasets from PhysGen resource (http://pga.mcw.edu) [17]. The package run time depends on a variety of factors including dataset size, computational resources, etc. Average analysis run time of the test datasets in our local environment was 1.34 seconds. The PhenStat is used as the main statistical software at the European Bioinformatics Institute (EBI) for the analysis of quality controlled data in the IMPC database available through the http://www.mousephenotype.org [18]. The package is also implemented at MRC Harwell for preliminary IMPC data analysis. The usage of PhenStat enables these analyses to be automated and version controlled.

## Methods

Phenotyping data collected at the Wellcome Trust Sanger Institute was approved by the Animal Welfare and Ethical review Board (AWERB) resulting in the approval licence: PPL 80/2076 Valid 27th Nov 2006—3rd Jan 2012; PPL 80/2485 valid 22nd Dev 2011—3rd Jan 2017. All efforts were made to minimize suffering by considerate housing and husbandry. Animal welfare was assessed routinely for all mice involved. Adult mice were killed by terminal anaesthesia followed by exsanguination and either cervical dislocation or removal of the heart.

In this work we present PhenStat, a novel software package of R, which identifies phenotypic associations from high throughput phenotyping experiments. The package depends on R and a number of other R packages listed in the usage section. Once R is installed the package by itself is operating system independent. Test data are provided in Supplementary Information: S1 Dataset and S2 Dataset.

### Structure of the package

**Fig 1:**
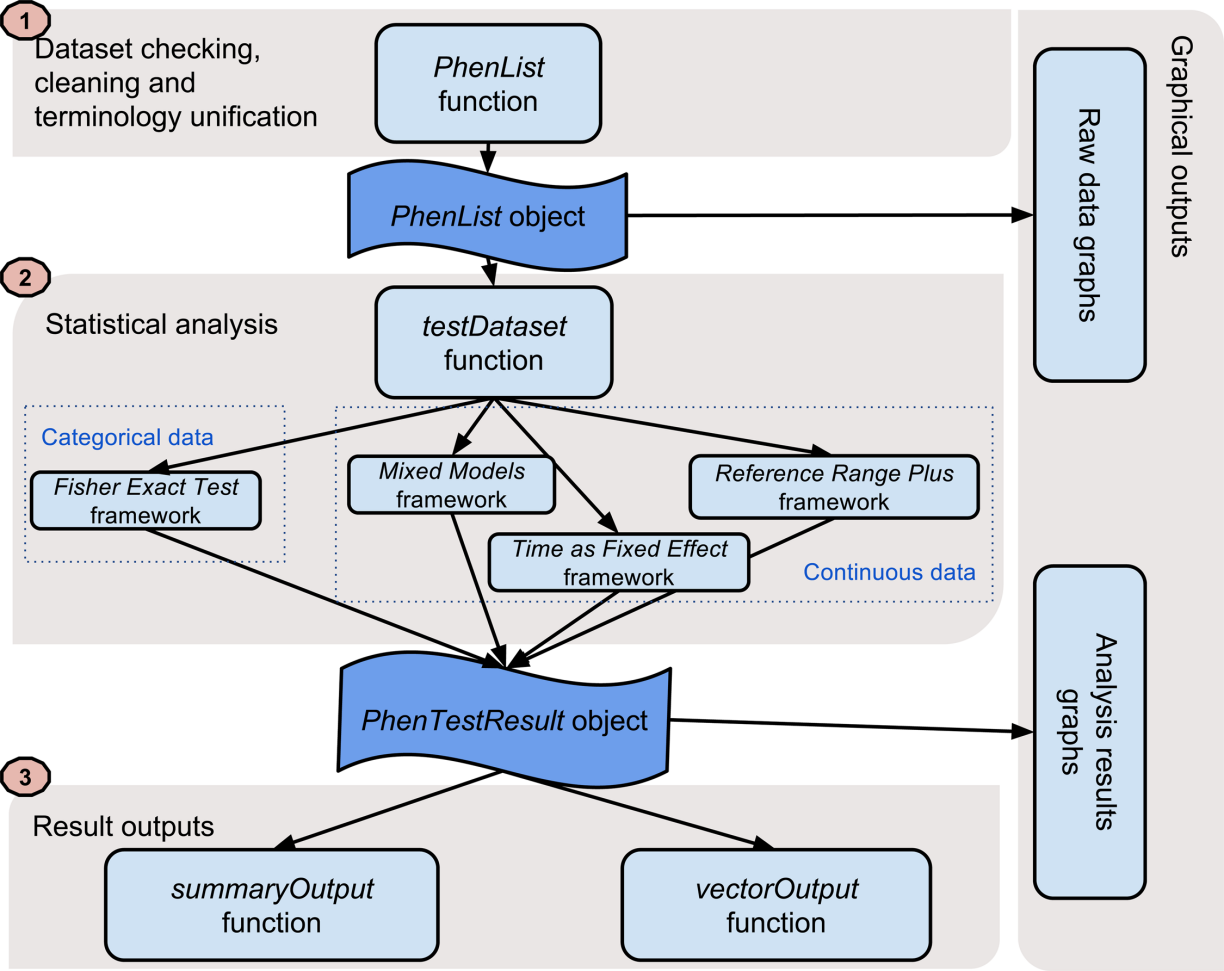
The PhenStat package's three-layer structure. The PhenStat package is designed with a three layer structure: dataset processing, analysis and result. In addition, there is a layer with graphical output.

For ease of use, the package consists of three layers as shown in Fig 1.

The first step, dataset checking and cleaning, prepares the data for analysis and is managed by the *PhenList* function and creates a *PhenList* object. Critical to this is a terminology unification procedure, necessary as different laboratories use different terms to describe the same object (e.g. “sex” versus “gender” in describing an animal’s sex). This step also includes a number of checking and cleaning processes to ensure the data are ready for downstream analysis. Any data cleaning steps performed (e.g. the removal of data) are reported.

Example of dataset processing:

> dataset_csv <- read.csv("myPhenDataset.csv")

> test <- PhenList(dataset_csv, testGenotype = "Sparc/Sparc",dataset.clean = TRUE)

The second step, statistical analysis, is managed by the *testDataset* function. This works as a manager for different statistical analyses methods and the “method” argument defines which method to use as discussed in the manuscript in “Statistical Methods Available”. The *testDataset* function performs basic checks that ensure the selected statistical analysis will be appropriate and successful: dependent variable presence, data variability checks, etc. If issues are identified, clear guidance is returned to the user. Results are stored in the *PhenTestResult* object regardless of the statistical method used. For all frameworks implemented, the statistical significance is assessed, the biological significance through an effect size is estimated and finally the genotype effect is classified e.g. "both sexes equally".

Example showing a test object obtained from the first step being analysed with the method ‘MM’ (Mixed Models method):

> result <- testDataset(test, depVariable = "Lean.Mass", method = "MM")

The final step, viewing the results, is provided by two functions depending on user needs. As shown in Fig 1, two functions, *summaryOutput* and *vectorOutput*, present numeric results to the user. These output formats were generated for differing user’s needs: *summaryOutput* for the interactive analysis of data and *vectorOutput* for large-scale application where automatic implementation would be required. The output vector is strictly defined and independent of analysis method that has been used.

Multiple graphical functions have been generated for visualization of the genotype effect and model diagnostics to assess quality of model estimations (e.g. the *qqplotGenotype* assesses the normality of the residuals). Further details on these functions, examples and interpretation can be found in the “Usage Example” section.

Examples of output functions:

> boxplotSexGenotype(test,"Weight","Body Weight")

> summaryOutput(result)

> qqplotGenotype(result)

### Statistical methods available

The selection of the statistical method is an important step in the process of phenotype data analysis and is dependent on the goal of the project, the experimental implementation, and the variable characteristics (e.g. continuous or categorical). Below we present the various methods we have implemented and explain for which workflows they are appropriate.

#### Categorical data—Fisher Exact Test method

The majority of categorical variables monitored in phenotyping studies are rare event classifications. For example: skull shape could be classified as normal or abnormal. With these small studies, it is critical that the abnormality is a rare event to give enough sensitivity to statistically detect differences between controls and treatment animals when a low number of treatment animals are phenotyped. To improve sensitivity, PhenStat uses all control data to assess the abnormality rate in the control population and takes no consideration of batch. Currently PhenStat uses the Fisher Exact Test [19], to assess for a statistically significant difference in the proportions observed between the knockout and control group for each sex.

#### Continuous data

Previous studies have found that sex, weight and batch were a significant source of variation for continuous variables [10,20] with batch encompassing other sources of variation, for example litter, cage and operator. While we could attempt to model all variation sources, we are limited by the number of covariates that can be included in a statistical model when the number of treatment animals is low (typically 3–7 animals for high-throughput pipelines). As such we have included differing methods in PhenStat to account for the most significant sources of variation when a model method is used.

#### Time as Fixed Effect method

Time as Fixed Effect (TF) approach is a regression method that models continuous data treating batch as a fixed effect. The method starts by deciding whether the user wish to include body weight (or other covariate representing weight e.g. body width, heart weight etc.) (Eq 2) or exclude (Eq 1) as a covariate in the starting model. The model is then optimised following an iterative top down mixed modelling strategy prior to assessing for a genotype effect. If batch is found to be significant, then the model estimates each batch effect to separate it from the potential genotype effect.

Variable=Genotype+Sex+Genotype*Sex+Batch Eq 1

Variable=Genotype+Sex+Genotype*Sex+Weight+Batch Eq 2

**Fig 2:**
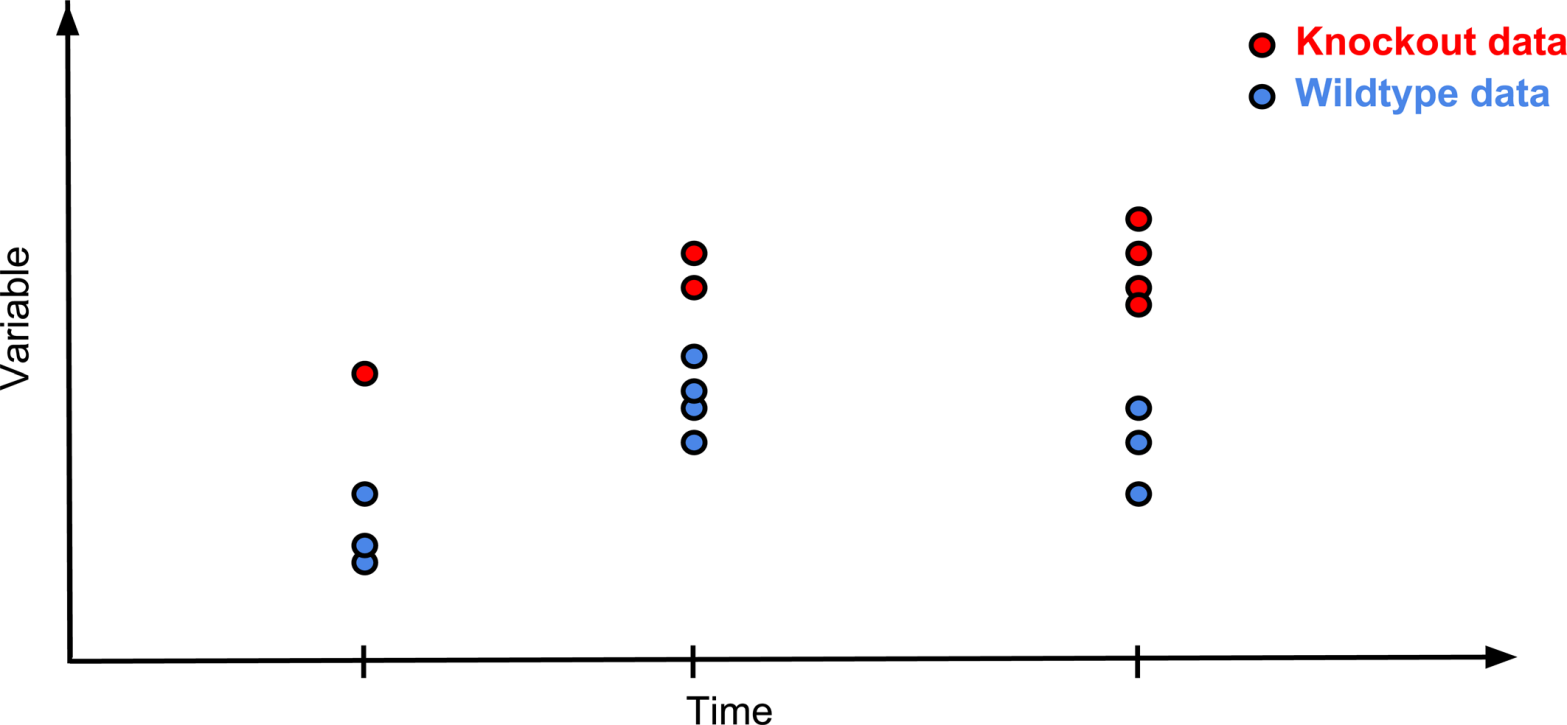
Graphical representation of a typical Time Fixed Effect data structure. The Time as a Fixed Effect analysis requires a data structure where there are multiple batches but each batch has concurrent controls.

This framework can be used in cases when there are up to five batches of treatment animals (e.g. knockout animals) and concurrent controls have been collected (Fig 2). Typically in high throughput studies the number of treated animals is limited in a concurrent design. That is why in the construct of the framework we limited the number of batches considered. The analysis requires removal of records that are not concurrent with treatment records or if treatment records lack concurrent controls. This is achieved by the *TFDataset* function, which will also report the data cleaning outcome impact on the data.

#### Mixed Model method

In the Mixed Model (MM) method, linear mixed models are used to describe the data with batch. Batch is treated as a random effect adding variation to the data that is assumed to be normally distributed. As with the TFE, an iterative top down mixed modelling strategy has been implemented to optimise the model prior to assessing for a genotype effect. Details of the implementation, including decision tree and models descriptions, are available in the PhenStat package user's guide (http://goo.gl/tfbA5k), and described in the literature [10,21]. There are two possible start models, depending on whether weight is included as a covariate (see Eq 3 and Eq 4).

Variable=Genotype+Sex+Genotype*Sex+(1|Batch) Eq 3

Variable=Genotype+Sex+Genotype*Sex+Weight+(1|Batch) Eq 4

If batch is not significant in explaining the variation, then the model optimisation results in a linear model being fitted to the data.

**Fig 3:**
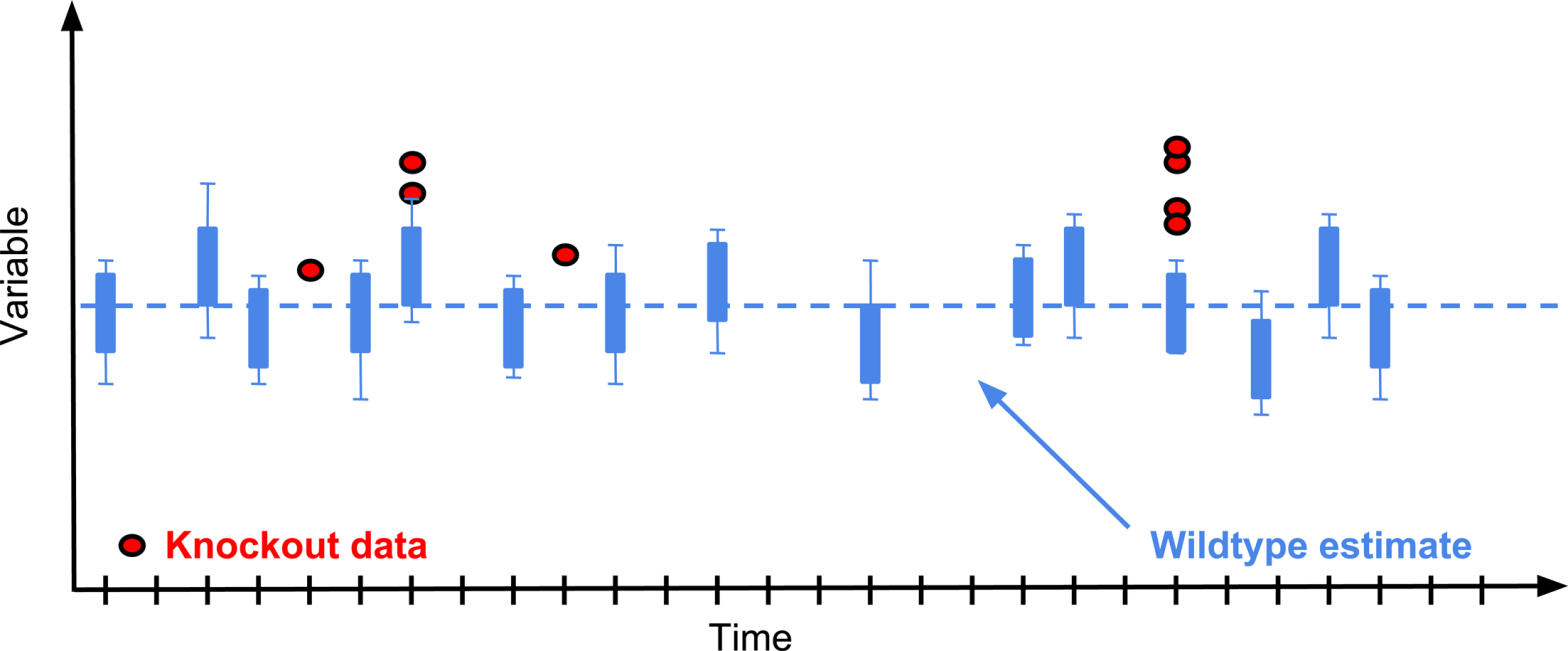
Graphical representation of a typical Mixed Model data structure. The Mixed Model analysis requires a data structure where there are multiple batches of knockout animals and regular batches of control animals. The analysis does not require concurrent controls.

This framework can be used in cases where both controls and treatment (e.g. knockout mice) are measured over multiple batches. The treatment animals do not have to be concurrent with controls (Fig 3).

#### Reference Range Plus method

**Fig 4:**
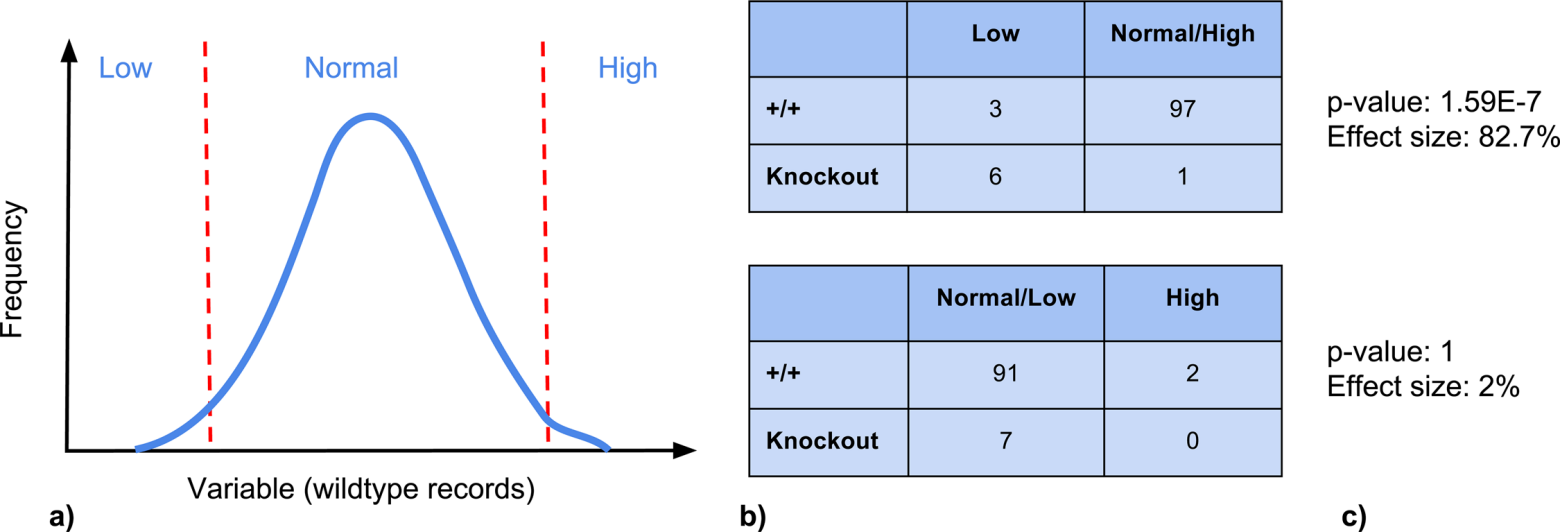
The Reference Range Plus method. **a)** Determining the thresholds for classifying the measures as low, normal or high based on the natural variation seen in the control data. **b)** Formation of a 2x2 count table following the classification of the measured animals. **c)** The observed proportions seen in tables (b) are compared with a Fisher Exact Test and effect quantified by calculating a change in penetrance in classification.

The "Reference Range Plus" (RR) method is an intuitive, simple, conservative method based on the concept that a significant phenotype can be called when the majority of animals lie outside the natural variation seen in the control animals within particular institute. A similar concept was used in the large scale study of knockout data from the Wellcome Trust Sanger Institute Mouse Genetics Project [15] and the ENU-mutagenesis project [22]. It is also comparable to medical investigations for humans where measurements are compared to baseline readings. Our implementation is based on classifying the analysable variable values as high, normal, low based on the natural variation seen within the control data and comparing the proportions seen with a Fisher Exact Test (Fig 4).

This framework can be used in studies where the other analysis pipelines are not suitable, for example in a study where it is a one batch design without concurrent controls, where the number of measures is low or where it has been difficult to design the experiment and as such there are significant variables beyond sex, batch and genotype that are varying.

### Assessing which method to use

Each analysis method has its own set of requirements and appropriate checks. Within the analysis step (*testDataset*), clear guidance is returned if analysis cannot be completed with the method selected. The *recommendMethod* function can be called prior to statistical analysis. It runs all the checks required by different methods and advises which analysis pipelines could be used.

> file <- system.file("extdata", "test1.csv", package = "PhenStat")

> test <- PhenList(dataset = read.csv(file),

testGenotype = "Sparc/Sparc")

> recommendMethod(test, depVariable = "Lean.Mass")

[1] "MM and RR"

As you can see from the example usage above, there are two methods suitable for this dataset/dependent variable combination: "Mixed Models" approach (MM) and "Reference Range Plus" approach (RR).

## Usage

The PhenStat package is a part of Bioconductor R package repository. The current release of Bioconductor is version 3.0; includes the latest version of PhenStat that is 2.0.1 and works with R version 3.1.1.

Installation steps:

1. First install the latest version of R
2. Download the latest version of PhenStat package from Bioconductor by starting R and entering the commands:

>source(“http://bioconductor.org/biocLite.R”)

>biocLite(“PhenStat”)

1. Load PhenStat package:

>libary(“PhenStat”)

An alternative to step 2, is to download the PhenStat tarball containing the source of the package directly from the GitHub repository where the latest development version of package is stored (http://goo.gl/8LV4VB) and to use the *install*.*package* command from R console:

>install.packages(<downloaded_file>, repos = NULL, type =“source”)

Please note, when installing an R package from source, any package dependencies have to be manually loaded. PhenStat depends on the following packages: “limma”, “methods”, “car”, “nlme”, “nortest”, “vcd”.

As input, the PhenStat requires a dataset presented as a data frame that can be stored in csv or txt file. PhenStat requires columns with information about genotypes and sexes to be present in the data frame in addition to the variable of interest. Columns with assay day (batch) and body weight data are desirable but not required.

### Usage example

We present a worked example using rat phenotyping data from the PhysGen phenotyping database (http://pga.mcw.edu/). This database contains both phenotyping data from various ENU strains and from a consomic project. In the consomic project, strains are compared where lines differ by one complete chromosome pair [23]. In this usage example, we will focus the analysis comparing the SS (Dahl Salt-Sensitive; SS/JrHsdMcwi) parent strain vs consomic strain SS-3^BN^/Mcwi (SS genomic background with a BN chromosome 3 introgressed) for the variable “peak contracture pressure”, which is one of the measurements of ischemic phenotypes from the cardiac protocol described in detail on the PhysGen web site. The goal is to detect an increase in intracavity pressure of 4 mmHg above end-diastolic values during an ischemic episode. Table 1 details the dataset and highlights the multi-batch nature common to high throughput phenotyping.

**Table 1:** Rat dataset characteristics for the peak contracture pressure variable of the cardiac study comparing SS and SS-3^BN^/Mcwi strains.

Genotype	Sex	Number of rats	Number of batches
SS (control)	Male	235	86
	Female	58	9
SS-3^BN^/Mcwi (treated)	Male	19	2
	Female	20	2

**Fig 5:**
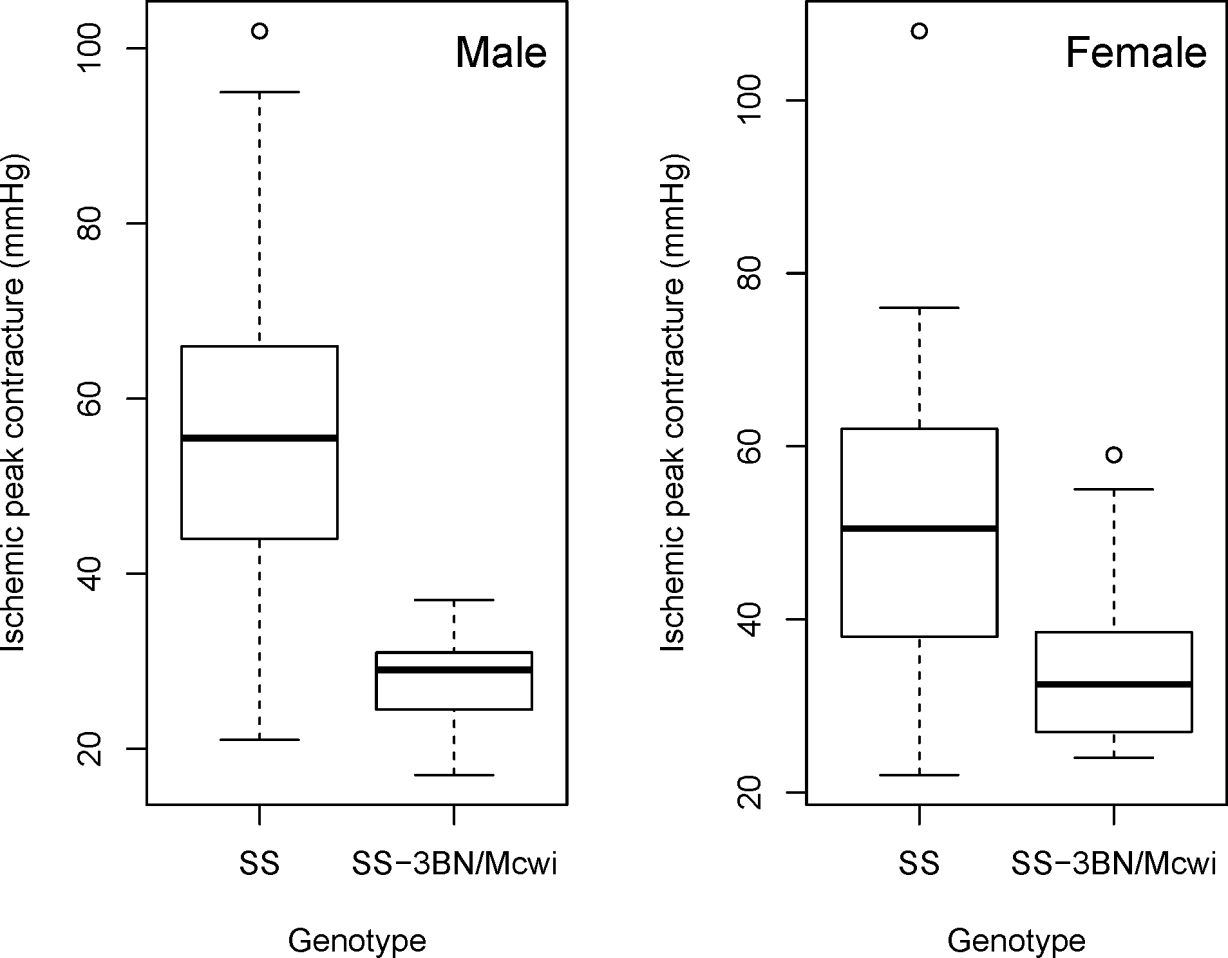
Example output of the PhenStat *boxplotGenotypeSex* function. Shown is the output from the *boxplotGenotypeSex* function obtained for the ischemic peak contracture pressure from a study on rats comparing SS strain to SS-3BN/Mcwi strain. Graphic highlights a visual difference in the variable of interest that could potentially be attributed to the genotype change.

**Fig 6:**
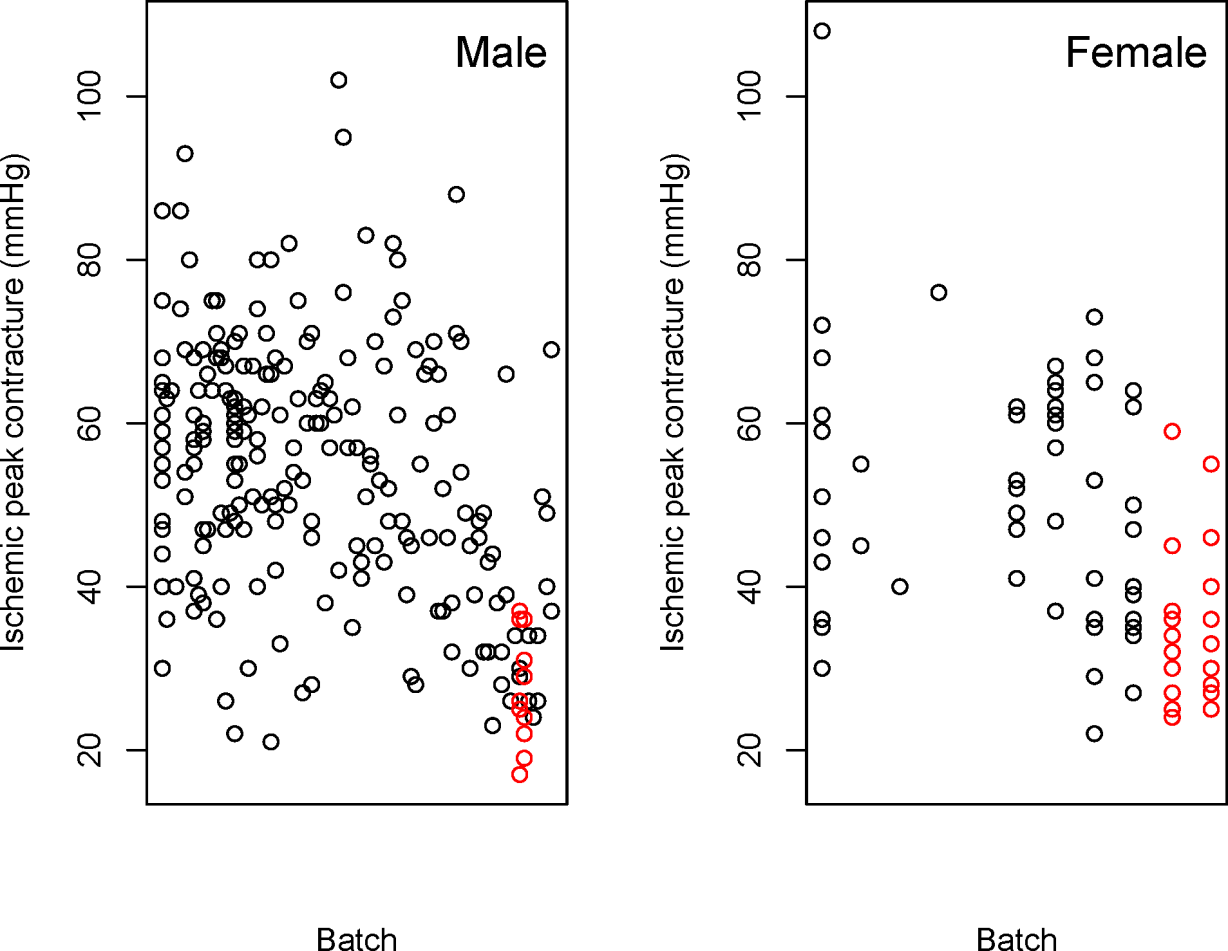
Example output of the PhenStat *scatterplotGenotypeSexBatch* function. Shown is the variation with batch in the peak contracture pressure readings for rats from SS strain (coloured in black) compared to the SS-3BN/Mcwi strain (coloured in red) visualised using the *scatterplotGenotypeSexBatch* function. This plot allows the user to visualize the batch variation and assess how the treatment effect compares to the observed batch variation. It is important to note that as dates can be entered in many forms, the batches are not ordered with time.

The dataset (S1 Dataset) was processed using PhenStat V2.0.1 and the code available in the Supplementary Information (S1 Code). Exploration of raw data (Fig 5 and Fig 6) highlights a visual difference in the variable of interest that could potentially be attributed to the genotype change.

The *recommendMethod* function indicates that that the data could be processed with the Mixed Model Method or the Reference Range Plus Method. Looking first at the conservative simple Reference Range Plus method, the analysis found a highly significant genotype effect as a high proportion of the rats were classified as low (Table 2) (male dataset, *p* value = 4.75e-6 with a 43% change in classification and female dataset *p* value = 4.64e-3 with a 26% change in classification).

**Table 2:** Reclassification of records from the rat dataset as low, normal and high relative to the natural variation (95%) in the control data.

Genotype	Sex	Low	Normal	High
SS	Males	8	204	6
	Female	2	48	2
SS-3^BN^/Mcwi	Males	13	22	0
	Female	6	14	0

**Fig 7:**
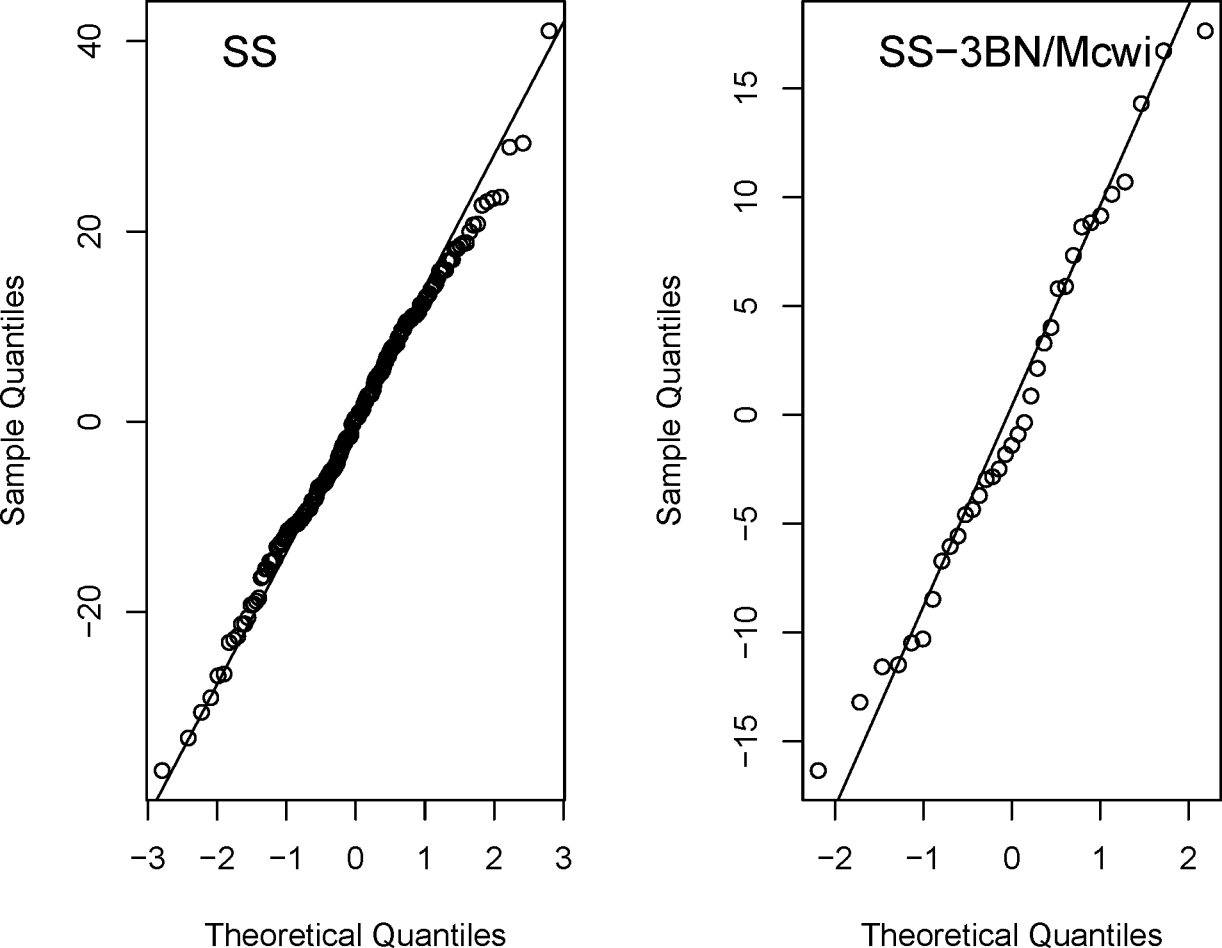
Example output of the PhenStat *qqplotGenotype* function. Data shown is the output from the *qqplotGenotype* function during the analysis of the ischemic peak contracture pressure from a study on rats comparing SS strain to SS-3BN/Mcwi strain when fitted with the Mixed Model method including body weight. This function allows an assessment of the model by examining the behaviour of the residuals, which is the difference between the measures and the model estimated values. A good model will have a normal distribution of residuals and the plot allows this to be assessed for each group being compared. Looking at the example, the residuals for both groups show no systematic deviations from the line indicating the model is fitting this data well.

Processing the same data using the Mixed Model method and excluding weight (Eq 3) completed a model optimization process and for this dataset, batch was not found to be significant source of variation, variance was found to be homogenous and the genotype effect was found to be sexual dimorphic as it depended on the sex of the animals. The final optimized model was used and it was found that there was a statistically significant genotype effect (p value = 9.92e-6) classified as sexual dimorphic as the effect was larger in the males (-26.65±2.44mmHg) than the females (-16.53±2.88 mmHg). A variety of diagnostics can be run to assess the model fit, for example Fig 7 assesses the residuals (differences between the measured and fitted) for normality.

**Fig 8:**
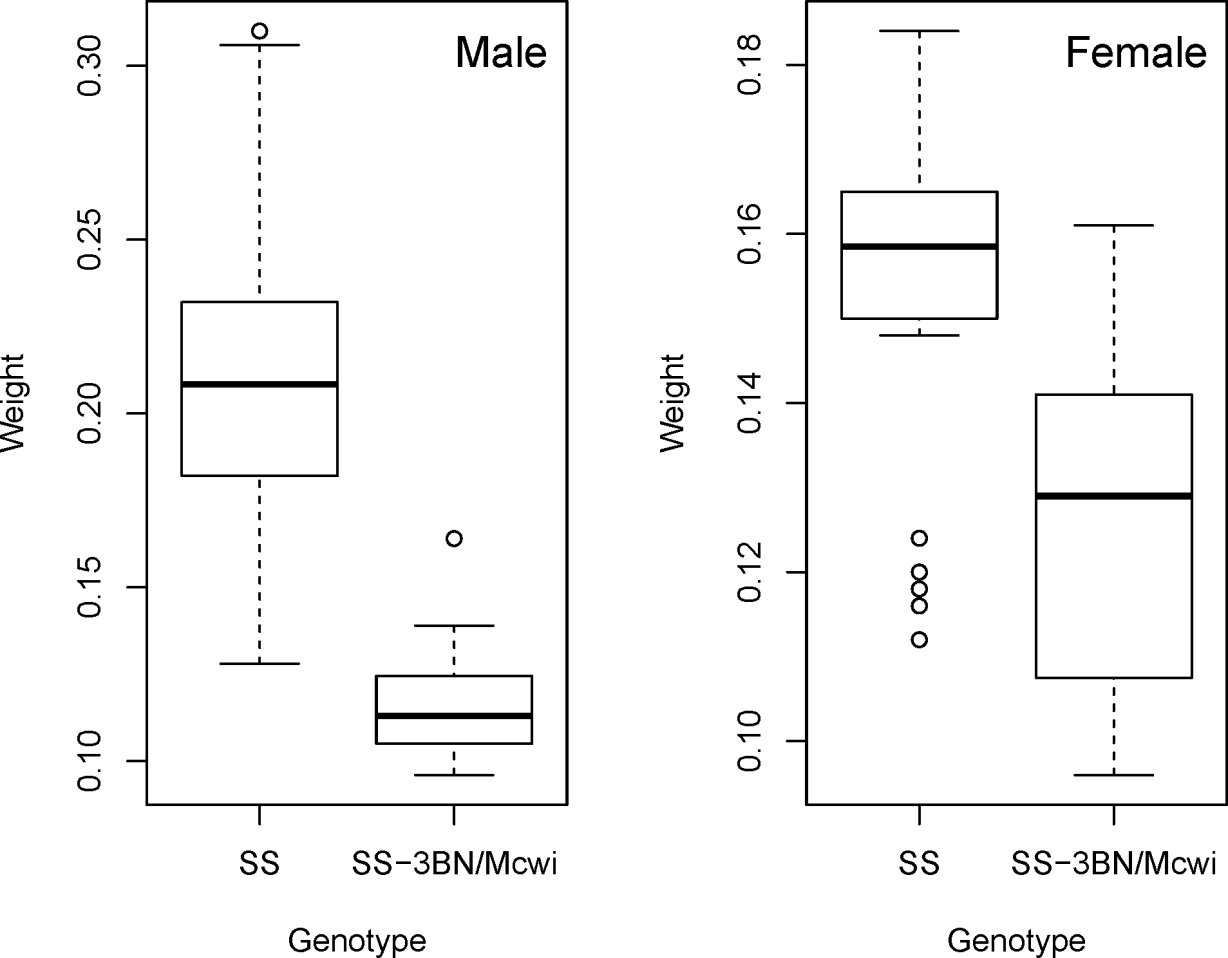
An example body weight phenotype. A graphically visualising of the body weight phenotyping observed in a study of the ischemic peak contracture pressure on rats comparing SS strain to SS-3BN/Mcwi strain.

**Fig 9:**
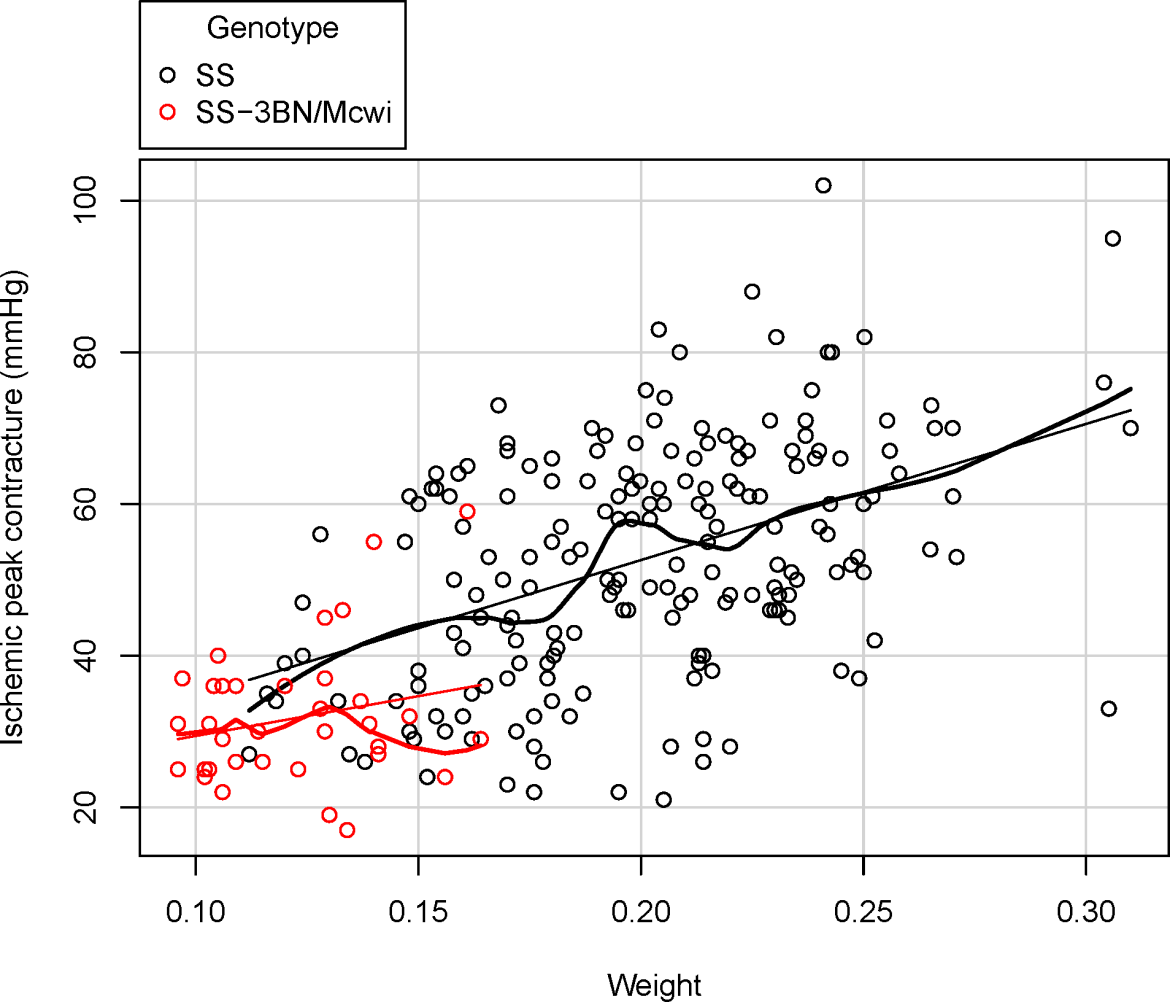
Example output of the PhenStat *scatterplotGenotypeWeight* function. Data shown is the output from the *scatterplotGenotypeWeight* function during analysis of the ischemic peak contracture pressure from a study on rats comparing SS strain to SS-3BN/Mcwi strain. Both a regression line and a loess line (locally weighted line) fitted for each genotype.

Alternatively, the Mixed Model method can be run to include a covariate to adjust for the animals’ weight (Eq 4). This is critical in phenotyping experiments, as body weight has been found to be a common phenotype with genotype alterations [24] and body size is a significant source of variation for many phenotyping variables [10,25]. Including a covariate for body weight can therefore increase the sensitivity of the study by accounting for more variation, or it can remove a confounding effect where the difference is arising solely from a body weight difference. Including body weight in the analysis, gives a model where batch was significant, the variances were heterogeneous across the genotype groups, and there was no evidence of sexual dimorphism. The final optimized model was used and it was found that there was no longer a statistically significant genotype effect (*p* value = 0.0959), the genotype differences was estimated at -6.23±3.73mmHg as the variation was now associated with body weight (*p* value = 4.08e-12). Looking at the body weight (Fig 8) we can see a large body weight phenotype particularly amongst the male rats, furthermore we can see that body weight correlates strongly with the variable of interest (Fig 9).

A further example usage with a mouse dataset from the IMPC project is shown in Supplementary Information S2 Code.

### Future work

We are testing a Biased Reduction Logistic Regression method [26] for inclusion in future releases of the PhenStat package which will add an assessment of sexual dimorphism for the categorical data.

We are also testing a transformation routine of continuous data based on Box-Cox power transform [27] to improve the model fit quality.

## Conclusion

Applying the appropriate statistical analysis is a challenge in assessing biological data [28–30] and is an area of active research for high throughput phenotyping [10,20]. There is a need for accessible, freely available statistical tools that support the community in choosing the best analysis, especially when complex statistical methods are involved. This supports reproducibility of results by other parties, which is important for all research, particularly for *in-vivo* studies.

PhenStat, an R package, has a variety of statistical analysis tools that have been developed based on known variation in experimental workflow and design of phenotyping pipelines used to identify phenotypic associations. PhenStat consists of many easy to use functions to perform and display statistical analysis of phenotyping data. The package provides a dataset processing function, statistical analysis processing which is easily adjustable for the different types of the analyses, and output functions that suit both interactive data analysis and large-scale database application.

The package requires minimal to no manual intervention, can be easily automated and hence can delivers a robust implementation of phenotype data processing. Additionally, PhenStat is the only R package to our knowledge that provides comprehensive Linear Mixed Models and Reference Range Plus implementation developed for the identification of phenotypic associations.

In comparison with the freely available InVivoStat [31] which focuses on traditional statistical approaches for animal experiments, PhenStat provides statistical methods specifically developed for high throughput phenotyping which are not available in InVivoStat (e.g. Linear Mixed Model method and Reference Range Plus method). The alternatives to PhenStat would be to use R directly, which would require deeper knowledge of R, and would lose the benefits of the version controlled analysis with known data cleaning steps.

PhenStat is a versioned package which can include new methods for the statistical analysis and output format by request. Full description of the PhenStat objects and functions, statistical analysis details, and usage examples, including cluster usage, are available in the PhenStat package user's guide (http://goo.gl/mKlX99).

## Supporting Information

S1 Code PhenStat demonstration on rat data (S1 Dataset). (DOCX)

S2 Code PhenStat demonstration on mouse data (S2 Dataset). (DOCX)

S1 Dataset Dataset containing rat phenotypic data. (CSV)

S2 Dataset Dataset containing mouse phenotypic data. (CSV)
