# Supplementary material for: PhenStat: A Tool Kit for Standardized Analysis of High Throughput Phenotypic Data
Source: PLoS One. 2015 Jul 6;10(7):e0131274. doi: 10.1371/journal.pone.0131274 (PMC4493137; doi:10.1371/journal.pone.0131274)
Supplement: S1 Code — (DOCX) [file pone.0131274.s001.docx]

# Code S1 - PhenStat demonstration on rat data

All commands in this code example are called from the R environment. Start the R program; on Windows and OS X (formerly known as Mac OS X), this usually mean double-clicking on the R application, on Unix-like system, type “R” at a shell prompt.

If the PhenStat package has not been yet installed then download the latest version of the PhenStat from Bioconductor by entering the commands:

> source(“http://bioconductor.org/biocLite.R”)

> biocLite(“PhenStat”)

Load PhenStat package:

> library(“PhenStat”)

## Dataset

Rat cardiac phenotypes data have been downloaded from the following URL: <http://pga.mcw.edu/pga2/phenotype/CARDIAC-M-ON.html?gender=GENDER_ON&value_type=MEAN&protocol=CARDIAC>

File with downloaded data has been saved under the name: "PhysGen_CARDIAC_consomic.csv" (also available as Dataset S1). This file contains all strains of rats including the ones to be analysed in this example: SS strain and comsomic SS-3^BN^/Mcwi strain:

> fileName <- "./PhysGen_CARDIAC_consomic.csv"

## PhenList object

The command line below creates the *PhenList* object “test” using the function *PhenList* which maps PhysGen CARDIAC protocol terminology to the PhenStat nomenclature; assigns test genotype and reference genotype; filters out records with other genotype values than test and reference genotypes.

> test <- PhenList(dataset=read.csv(fileName),

testGenotype="SS-3BN/Mcwi",

refGenotype="SS",

dataset.colname.genotype="STRAIN",

dataset.colname.sex="GENDER",

dataset.values.male="M",

dataset.values.female="F",

dataset.colname.batch="RAT_COHORT",

dataset.colname.weight="body.weight..kg.")

Warning:

Dataset has been cleaned by filtering out records with genotype value other than test genotype 'SS-3BN/Mcwi' or reference genotype 'SS'.

Information:

Dataset's 'Genotype' column has following values: 'SS', 'SS-3BN/Mcwi'

Information:

Dataset's 'Sex' column has following value(s): 'Female', 'Male'

## Dataset graphics

There are raw data graphic functions available in PhenStat that allows the user to explore the dataset before the actual statistical analysis. Function’s *boxplotSexGenotype* result is shown in Figure C1.1 and Figure C1.3 for ischemic protocol variable ischemic peak contracture pressure and for the body weight of rats appropriately.

> boxplotSexGenotype(test,

depVariable="ischemic.peak.contracture..mmHg.",

graphingName="Ischemic peak contracture (mmHg)")

**Figure C1.1**: Example output of the PhenStat *boxplotGenotypeSex* function. Shown is the output obtained for the ischemic peak contracture pressure from a study on rats comparing SS strain to SS-3^BN^/Mcwi strain.

Function’s *scatterplotSexGenotypeBatch* result is shown in Figure C1.2.

>scatterplotSexGenotypeBatch(test, depVariable="ischemic.peak.contracture..mmHg.",

graphingName="Ischemic peak contracture (mmHg)")

**Figure C1.2**: Example output of the PhenStat *scatterplotGenotypeSexBatch* function. Shown is the variation with batch in the peak contracture pressure readings for rats from SS strain (coloured in black) compared to the SS-3^BN^/Mcwi strain (colored in red). This plot allows the user to visualise the batch variation and assess how the treatment effect compares to the observed batch variation. It is important to note that as dates can be entered in many forms, the batches are not ordered with time.

> boxplotSexGenotype(test, depVariable="Weight")

**Figure C1.3**: Visualising the body weight phenotyping observed in a study of the ischemic peak contracture pressure on rats comparing SS strain to SS-3^BN^/Mcwi strain.

Function’s *scatterplotSexGenotypeWeight* result is shown in Figure C1.4.

> scatterplotGenotypeWeight(test,

depVariable="ischemic.peak.contracture..mmHg.",

graphingName="Ischemic peak contracture (mmHg)")

**Figure C1.4**: Example output of the PhenStat *scatterplotGenotypeWeight* function. Data shown is the output from analysis of the ischemic peak contracture pressure from a study on rats comparing SS strain to SS-3^BN^/Mcwi strain. Both a regression line and a loess line (locally weighted line) fitted for each genotype.

Figure C1.1 and Figure C1.2 highlights a visual difference in the variable of interest that could potentially be attributed to the genotype change. Looking at the body weight (Figure C1.3) we can see a large body weight phenotype particularly amongst the male rats, furthermore we can see that body weight correlates strongly with the variable of interest (Figure C1.4).

## Recommend appropriate analysis method

The Function *recommendMethod* returns the statistical analysis methods suitable for the dataset and variable of interest. Recommended methods for the rat dataset and ischemic protocol’s measurement ischemic peak contracture pressure are Mixed Model method (MM) and Reference Range Plus method (RR).

> recommendMethod(test,depVariable="ischemic.peak.contracture..mmHg.")

[1] "MM and RR"

## Statistical analysis

Reference Range Plus method is called using function *testDataset* with the argument “method” equal set to “RR”. In this example the argument “RR_controlPointsThreshold” is set to 50 since the default value (60) is too restrictive for this particular dataset. The output of the *testDataset* function is *PhenTestResult* object called “resultRR”.

> resultRR <- testDataset(phenList = test,

depVariable="ischemic.peak.contracture..mmHg.",

method="RR",RR_controlPointsThreshold=50)

Information:

Dependent variable: 'ischemic.peak.contracture..mmHg.'.

Information:

Method: Reference Ranges Plus framework.

Function *summaryOutput* returns the analysis results including classification tag and effect sizes.

> summaryOutput(resultRR)

Test for dependent variable:

*** ischemic.peak.contracture..mmHg. ***

Method:

*** Reference Ranges Plus framework ***

----------------------------------------------------------------------------

Model Output ('*' highlights results with p-values less than threshold 0.01)

----------------------------------------------------------------------------

                                   All    Males only Females only

* Low classification p-value:      0.0000 0.0000     0.0093

* Low classification effect size:  33%    43%        26%

 High classification p-value:      1.0000 1.0000     1.0000

 High classification effect size: 3%     3%         4%

----------------------------------------------------------------------------

Classification Tag

----------------------------------------------------------------------------

With phenotype threshold value 0.01 - significant in males (Low), females (Low) and in combined dataset (Low)

----------------------------------------------------------------------------

Thresholds

----------------------------------------------------------------------------

p-value threshold:            0.01

Natural variation:            95

Min control points:           50

Normal values 'males only':   26.000 to 84.725

Normal values 'females only': 27.550 to 75.175

----------------------------------------------------------------------------

Count Matrices

----------------------------------------------------------------------------

'All' matrix:

       SS SS-3BN/Mcwi

Low     10          13

Normal 252          22

High     8           0

'Males only' matrix:

       SS SS-3BN/Mcwi

Low      8           7

Normal 204           8

High     6           0

'Females only' matrix:

      SS SS-3BN/Mcwi

Low     2           6

Normal 48          14

High    2           0

The results of RR indicate genotype effect to be significant in males, females and combined dataset (males and females together) due to a movement in classification towards “Low”.

The output of the RR method can be visualized using function *categoricalBarplot*:

> categoricalBarplot(resultRR)

**Figure C1.5**: Example output of PhenStat *categoricalBarplot* function. Function visualises the categorical data formed from the RR framework as summary percentage data. It reports the percentage of each classification observed for up to three datasets: all data, male only and female only. It is important to note that percentage accuracy is very dependent on the number of readings so it is important to consider the dataset size when interpreting these graphs. Therefore tables showing both the percentage and count values are included in the *summaryOutput*.

For the second recommended method, which is Mixed Model method, there are two options: include animal body size (weight) as a covariant (*testDataset* function’s argument “equation” equals to ”withWeight”, which is the default argument value) or exclude weight from the model (equation=”withoutWeight”).

> resultWithoutWeight<-testDataset(test,

depVariable="ischemic.peak.contracture..mmHg.",

method="MM",

equation="withoutWeight")

Information:

Dependent variable: 'ischemic.peak.contracture..mmHg.'.

Information:

Perform all MM framework stages: startModel and finalModel.

Information:

**Method: Mixed Model framework.**

Information:

**Equation: 'withoutWeight'.**

Information:

Calculated values for model effects are: keepBatch=FALSE, keepEqualVariance=FALSE, keepWeight=FALSE, keepSex=FALSE, keepInteraction=TRUE.

The function *summaryOutput* allows the user to see a summary of the results on the screen. It was found that there was a statistically significant genotype effect (p value=9.92e-6) classified as sexual dimorphic as the effect was larger in the males (-26.65±2.44mmHg) than the females (-16.53±2.88 mmHg).

> summaryOutput(resultWithoutWeight)

Test for dependent variable:

*** ischemic.peak.contracture..mmHg. ***

Method:

*** Mixed Model framework ***

----------------------------------------------------------------------------

Model Output

----------------------------------------------------------------------------

Genotype effect: 0.0000

Final fitted model: ischemic.peak.contracture..mmHg. ~ Sex + Genotype:Sex

Was batch significant? FALSE

Was variance equal? FALSE

Was there evidence of sexual dimorphism? yes (p-value 0.0076)

Genotype percentage change Female: -32.27%

Genotype percentage change Male: -48.83%

----------------------------------------------------------------------------

Classification Tag

----------------------------------------------------------------------------

With phenotype threshold value 0.01 - different size as males greater

----------------------------------------------------------------------------

Model Output Summary

----------------------------------------------------------------------------

                                  Value Std.Error    t-value p-value

(Intercept)                    51.230769  2.153181  23.793067 3.964722e-71

SexMale                         3.360974  2.396261   1.402591 1.617694e-01

SexFemale:GenotypeSS-3BN/Mcwi -16.530769  2.875449  -5.748935 2.202909e-08

SexMale:GenotypeSS-3BN/Mcwi   -26.658410  2.438970 -10.930193 1.214144e-23

Alternatively, the Mixed Model method can be run to include a covariate to adjust for the animals’ body size.

> resultWithWeight<-testDataset(test,

depVariable="ischemic.peak.contracture..mmHg.",

method="MM")

Information:

Dependent variable: 'ischemic.peak.contracture..mmHg.'.

Information:

Perform all MM framework stages: startModel and finalModel.

Information:

**Method: Mixed Model framework.**

Information:

**Equation: 'withWeight'.**

Information:

Calculated values for model effects are: keepBatch=TRUE, keepEqualVariance=FALSE, keepWeight=TRUE, keepSex=TRUE, keepInteraction=FALSE.

When we use the *summaryOutput* function to see the modelling result, we find that there was no longer a statistically significant genotype effect (*p* value=0.0959) as the genotype differences was estimated at -6.23±3.73mmHg as the variation is now associated with body weight (*p* value 4.08e-12, 202.89±26.88mmHg). Looking at the body weight (Figure C1.3) we can see a large body weight phenotype particularly amongst the male rats, furthermore we can see that body weight correlates strongly with the variable of interest (Figure C1.4). This explains the sexual dimorphic call in the model without weight as the large body weight differences specific to the males lead to a large difference in the variable of interest.

> summaryOutput(resultWithWeight)

Test for dependent variable:

*** ischemic.peak.contracture..mmHg. ***

Method:

*** Mixed Model framework ***

----------------------------------------------------------------------------

Model Output

----------------------------------------------------------------------------

Genotype effect: 0.0959

Final fitted model: ischemic.peak.contracture..mmHg. ~ Genotype + Sex + Weight

Was batch significant? TRUE

Was variance equal? FALSE

Was there evidence of sexual dimorphism? no (p-value 0.6102)

Genotype percentage change Female: -12.18%

Genotype percentage change Male: -11.43%

----------------------------------------------------------------------------

Classification Tag

----------------------------------------------------------------------------

With phenotype threshold value 0.01 - no significant change

----------------------------------------------------------------------------

Model Output Summary

----------------------------------------------------------------------------

                        Value Std.Error  DF   t-value      p-value

(Intercept)          16.238952  5.535147 149  2.933789 3.878600e-03

GenotypeSS-3BN/Mcwi  -6.238318  3.739489 149 -1.668227 9.737078e-02

SexMale              -5.673022  2.262239 149 -2.507702 1.322380e-02

Weight              202.891398 26.881104 149  7.547733 4.080643e-12

A variety of diagnostics can be run to assess the quality of the model fit, for example *qqplotGenotype* function generates graphic which examines the distribution of residuals (differences between the observed values and estimated values) (Figure C1.6).

> qqplotGenotype(resultWithWeight)

**Figure C1.6**: Example output of the PhenStat *qqplotGenotype* function. Data shown is the output from analysis of the ischemic peak contracture pressure from a study on rats comparing SS strain to SS-3^BN^/Mcwi strain when fitted with the mixed model method including body weight. This function allows an assessment of the model; by examining the behavior of the residuals defined as the differences between the measures and the model estimated values. Looking at the example, the residuals for both groups show no systematic deviations from the line indicating the model is fitting this data well.

In addition to the *qqplotGenotype* function, there are other graphical tools to assess model fit. The function *plotResidualPredicted* produces a graphic to assess the distribution of the residual along the predicted values allowing the user to assess the model fit along for different signal strength (Figure C.1.7). The function *qqplotRandomEffect* (Figure C.18) and *qqplotRotatedResiduals* (Figure C1.9) allows the user to assess the assumption of a normal distribution of batch. Finally, the *boxplotResidualsBatch* function (Figure C1.10) allows the user to assess if any particular batch is poorly represented by the model. These model diagnostic functions indicate that the rat dataset has been well fitted by the model.

> plotResidualPredicted(resultWithWeight)

**Figure C1.7**: Example output of the PhenStat *plotResidualPredicted* function. This function plots the residuals against the predicted readings for both genotypes. The predicted readings are the values the model would estimate for the variable of interest. Looking at the rat data, we can see that there spread of the residuals is fairly consistent, however there are some data points that are not being fit well by the model.

> qqplotRandomEffects(resultWithWeight)

**Figure C1.8**: Example output of the PhenStat *qqplotRandomEffects* function. This function is assessing the assumption that the batch effects are normally distributed. The estimates of the random effects, aka the estimates of the batch effects in this scenario, are called best linear unbiased prediction BLUPs. Here a normal Q-Q plot is used to plot the estimated BLUPs against a normal distribution. So looking at the rat example, the majority of the data points are distributed along the line. There is some systematic deviation at the tails but it is a small percentage of the points and we can conclude the distribution is not too far from the ideal and the model is a good representation of the data.

> qqplotRotatedResiduals(resultWithWeight)

**Figure C1.9**: Example output of the PhenStat *qqplotRotatedResiduals* function. This function, allows the user to consider the normality of the “rotated” and “unrotated” residuals. Looking at the rat example, the majority of the data points are distributed along the line so we can conclude the distribution is not too far from the ideal and the model is a good representation of the data.

> boxplotResidualsBatch(resultWithWeight)

**Figure C1.10**: Example output of the PhenStat *boxplotResidualsBatch* function. This function allows visualisation to assist the user to assess whether the deviation in the residual is consistent across all the batches and similar in size between the two groups. For the rat example, we can see that the variation in residual is consistent across all the batches and similar in size between the genotype groups.
