# Supplementary material for: PhenStat: A Tool Kit for Standardized Analysis of High Throughput Phenotypic Data
Source: PLoS One. 2015 Jul 6;10(7):e0131274. doi: 10.1371/journal.pone.0131274 (PMC4493137; doi:10.1371/journal.pone.0131274)
Supplement: S2 Code — (DOCX) [file pone.0131274.s002.docx]

# Code S2 - PhenStat demonstration on mouse data

All commands in this code example are called from the R environment. Start the R program; on Windows and OS X (formerly known as Mac OS X), this usually mean double-clicking on the R application, on Unix-like system, type “R” at a shell prompt.

If the PhenStat package has not been yet installed then download the latest version of the PhenStat from Bioconductor by entering the commands:

> source(“http://bioconductor.org/biocLite.R”)

> biocLite(“PhenStat”)

Load PhenStat package:

> library(“PhenStat”)

## Dataset

The package IMPCdata, which is also available in Bioconductor allows ready-for-analysis dataset retrieval directly from the IMPC (International Mouse Phenotyping Consortium) database.

If the IMPCdata package has not been yet installed then download the latest version of the IMPCdata from Bioconductor by entering the commands:

> source(“http://bioconductor.org/biocLite.R”)

> biocLite(“IMPCdata”)

Load IMPCdata package:

> library(“IMPCdata”)

> IMPC_dataset <- getIMPCDataset("WTSI","MGP_001","IMPC_CBC_001",

                               "IMPC_CBC_003_001","MGI:4431644")

The IMPC dataset that has been retrieved for a specified combination of phenotyping center (WTSI), pipeline ("MGP_001" - MGP Select Pipeline), procedure ("IMPC_CBC_001" - Clinical Blood Chemistry), parameter ("IMPC_CBC_003_001" - Chloride) and allele ("MGI:4431644" - *Fbxo47*<tm1a(EUCOMM)Wtsi>).

Table S2.1 details the dataset and highlights the multi-batch nature common to high throughput phenotyping.

| **Genotype** | **Sex** | **Number of mice** | **Number of batches** |
| --- | --- | --- | --- |
| +/+ (control) | Male | 1322 | 109 |
|  | Female | 1370 | 109 |
| MDTZ (treated) | Male | 14 | 5 |
|  | Female | 14 | 6 |

**Table S2.1**: Dataset characteristics for the chloride variable of the IMPC Clinical Blood Chemistry study from WTSI comparing wild type mice (“+/+”) against mice with knocked out *Fbxo47* gene (MDTZ).

## PhenList object

In the datasets retrieved from the IMPC the genotype values are stored in the column called “Colony”:

> levels(IMPC_dataset$Colony)

[1] "+/+" "MDTZ"

The command line below creates the *PhenList* object “testIMPC” using the function *PhenList* which maps IMPC terminology to the PhenStat nomenclature; assigns test genotype and reference genotype.

> testIMPC <- PhenList(dataset=IMPC_dataset,

                     testGenotype="MDTZ",

                     refGenotype="+/+",

                     dataset.colname.genotype="Colony")

## Dataset graphics

 There are raw data graphic functions available in PhenStat that allows the user to explore the dataset before the actual statistical analysis. Function’s *boxplotSexGenotype* result is shown in Figure C2.1 for chloride measurements.

> boxplotSexGenotype(testIMPC, depVariable="Value", graphingName="Chloride")

**Figure C2.1**: Example output of the PhenStat *boxplotGenotypeSex* function. Shown is the output obtained for the chloride from a study on IMPC dataset comparing wild type animals to mice with knocked out *Fbxo47* gene (MDTZ).

>scatterplotSexGenotypeBatch(testIMPC, depVariable="Value", graphingName="Chloride")

**Figure C2.2**: Example output of the PhenStat *scatterplotGenotypeSexBatch* function. Shown is the variation with batch in the chloride readings for mice from wild type strain (coloured in black) compared to the knockout strain (colored in red). This plot allows the user to visualise the batch variation and assess how the treatment effect compares to the observed batch variation. It is important to note that as dates can be entered in many forms, the batches are not ordered with time.

Function’s *scatterplotSexGenotypeBatch* result is shown in Figure C2.2.

## Recommend appropriate analysis method

Function *recommendMethod* returns the statistical analysis methods suitable for the dataset and variable of interest. Recommended methods for IMPC dataset and chloride measurements are Time as Fixed Effect method (TF), Mixed Model method (MM) and Reference Range Plus method (RR).

> recommendMethod(testIMPC,"Value")

[1] "TF, MM and RR"

## Statistical analysis

For the TF method, where batch is treated as a fixed effect, only knockout animals with concurrent controls can be processed. Therefore, before PhenStat can analyse data using TF method, a dataset has to be filtered such that only knockout animals with concurrent controls remain. Function *TFDataset* cleans dataset by filtering out inappropriate data. The output will also give information on the cleaning process followed.

> testIMPC_TF <- TFDataset(testIMPC,depVariable = "Value")

Data points containing 'Value' by batch levels:

|  ----------- |  ----------- |  ----------- |  ----------- | -------------|

|              |          +/+ |          +/+ |         MDTZ |         MDTZ |

|  ----------- |  ----------- |  ----------- |  ----------- | -------------|

|        Batch |       Female |         Male |       Female |         Male |

|  ----------- |  ----------- |  ----------- |  ----------- | -------------|

| * 01/05/2012 |           14 |           14 |            0 |            0 |

|  ----------- |  ----------- |  ----------- |  ----------- | -------------|

| * 01/05/2013 |           12 |           10 |            0 |            0 |

|  ----------- |  ----------- |  ----------- |  ----------- | -------------|

| * 02/01/2014 |            0 |            4 |            0 |            0 |

|  ----------- |  ----------- |  ----------- |  ----------- | -------------|

| * 02/04/2012 |           14 |           14 |            0 |            0 |

|  ----------- |  ----------- |  ----------- |  ----------- | -------------|

| * 02/05/2012 |            0 |            0 |            0 |            2 |

|  ----------- |  ----------- |  ----------- |  ----------- | -------------|

| * 02/07/2012 |           12 |           14 |            0 |            0 |

|  ----------- |  ----------- |  ----------- |  ----------- | -------------|

...

| * 29/07/2013 |           14 |           14 |            0 |            0 |

|  ----------- |  ----------- |  ----------- |  ----------- | -------------|

|   30/05/2012 |           12 |           12 |            1 |            1 |

|  ----------- |  ----------- |  ----------- |  ----------- | -------------|

| * 30/05/2013 |           14 |           14 |            0 |            0 |

|  ----------- |  ----------- |  ----------- |  ----------- | -------------|

| * 30/10/2012 |           10 |           14 |            0 |            0 |

|  ----------- |  ----------- |  ----------- |  ----------- | -------------|

| * 30/10/2013 |           14 |           14 |            0 |            0 |

|  ----------- |  ----------- |  ----------- |  ----------- | -------------|

| * 31/07/2012 |           14 |           14 |            0 |            0 |

|  ----------- |  ----------- |  ----------- |  ----------- | -------------|

* - removed record(s)

Number of batch levels left: 5

Records removed (reference genotype): 94%

Records removed (test genotype): 0%

Functions *boxplotSexGenotype* (Figure C2.3) and *scatterplotSexGenotypeBatch* (Figure 2.4) help to visualise the results of the cleaning procedure.

> boxplotSexGenotype(testIMPC_TF, depVariable="Value", graphingName="Chloride")

**Figure C2.3**: Example output of the PhenStat *boxplotGenotypeSex* function. Shown is the output obtained for the plasma chemistry chloride measure, after the *TFDataset* function has cleaned the original dataset, for a study comparing wild type animals to mice with knocked out *Fbxo47* gene (MDTZ).

> scatterplotSexGenotypeBatch(testIMPC_TF, depVariable="Value",

graphingName="Chloride")

**Figure C2.4**: Example output of the PhenStat *scatterplotGenotypeSexBatch* function. Shown is the variation with batch in the chloride readings for mice from wild type (coloured in black) compared to the knockout (colored in red) after the function *TFDataset* has cleaned the original IMPC dataset.

The dataset is cleaned and ready for the TF method that can be called from the function *testDataset* with the argument “method” set to “TF”. Please notice that the IMPC dataset from the IMPCdata package doesn’t include weight records for animals and the regression methods (TF, MM) are run with equation “withoutWeight” automatically.

> resultIMPC_TF<-testDataset(testIMPC_TF, depVariable = "Value", method="TF" )

Warning:

Weight column is not present in dataset.

Equation 'withWeight' can't be used in such a case and has been replaced to 'withoutWeight'.

Information:

Dependent variable: 'Value'.

Information:

Perform all TF framework stages: startTFModel and finalTFModel.

Information:

**Method: Time as Fixed Effect framework.**

Information:

**Equation: 'withoutWeight'.**

Information:

Calculated values for model effects are: keepBatch=TRUE, keepEqualVariance=TRUE, keepWeight=FALSE, keepSex=TRUE, keepInteraction=FALSE.

Function *summaryOutput* returns summary of the modeling results.

> summaryOutput(resultIMPC_TF)

Test for dependent variable:

*** Value ***

Method:

*** Time as Fixed Effect framework ***

----------------------------------------------------------------------------

Model Output

----------------------------------------------------------------------------

Genotype effect: 0.0870

Final fitted model: Value ~ Genotype + Sex + Batch

Was batch significant? TRUE

Was variance equal? TRUE

Was there evidence of sexual dimorphism? no (p-value 0.4208)

Genotype percentage change Female: 0.52%

Genotype percentage change Male: 0.52%

----------------------------------------------------------------------------

Classification Tag

----------------------------------------------------------------------------

With phenotype threshold value 0.01 - no significant change

----------------------------------------------------------------------------

Model Output Summary

----------------------------------------------------------------------------

                     Value Std.Error    t-value       p-value

(Intercept)     107.1693742 0.2650980 404.263204 1.643178e-224

GenotypeMDTZ      0.5533852 0.3294215   1.679870  9.512127e-02

SexMale          -1.4474490 0.2188910  -6.612649  6.648367e-10

Batch20/08/2012   2.6011940 0.3408440   7.631625  2.764515e-12

Batch24/04/2012   2.1072116 0.3303679   6.378378  2.222731e-09

Batch28/08/2012   3.1482431 0.3451904   9.120308  5.482083e-16

Batch30/05/2012   3.3079361 0.3523550   9.388078  1.129856e-16

From the output we can see that the genotype effect is not significant according to TF method (p value=0.0870).

Model diagnostic functions assisting the user to assess the quality of the fitted model: *qqplotGenotype* function’s results are shown in Figure C2.5, *plotResidualsPredicted* function’s results are shown in Figure C2.6 and function’s *boxplotResidualsBatch* results are in Figure C2.7.

> qqplotGenotype(resultIMPC_TF)

**Figure C2.5**: Example output of the PhenStat *qqplotGenotype* function. Data shown is the output from analysis of the chloride clinical chemistry measure from a study on mice comparing wild type strain to knockout MDTX when fitted with the time as fixed effect method. Looking at the example, the residuals on both groups have a small systematic pattern in the deviations at the tails but the effect is minor and a small proportion of the data indicating the model is a good representation of the data.

> plotResidualPredicted(resultIMPC_TF)

**Figure C2.6**: Example output of the PhenStat *plotResidualPredicted* function. This function plots the residuals against the predicted readings for both genotypes. The predicted readings are the values the model would estimate for the variable of interest. Looking at the data, we can see that the spread of the residuals is fairly consistent, suggesting the model is a good fit for all data points.

> boxplotResidualsBatch(resultWithWeight)

**Figure C2.7**: Example output of the PhenStat *boxplotResidualsBatch* function. This function allows visualisation to assist the user to assess whether the deviation in the residual is consistent across all the batches and similar in size between the two groups. For the mice example, we can see that the variation in residual is fairly consistent across all the batches. The batch effect is significant especially in the knockout group (MDTZ).

The next method recommended was RR. As with other methods, the analysis methods RR is run from the *testDataset* function setting the argument “method” to “RR”.

> resultIMPC_RR<-testDataset(testIMPC,depVariable = "Value", method="RR" )

Information:

Dependent variable: 'Value'.

Information:

**Method: Reference Ranges Plus framework.**

> summaryOutput(resultIMPC_RR)

Test for dependent variable:

*** Value ***

Method:

*** Reference Ranges Plus framework ***

----------------------------------------------------------------------------

Model Output ('*' highlights results with p-values less than threshold 0.01)

----------------------------------------------------------------------------

                                  All    Males only Females only

 Low classification p-value:      0.3369 1.0000     0.0984

 Low classification effect size:  4%     3%         12%

 High classification p-value:     1.0000 1.0000     1.0000

 High classification effect size: 3%     3%         3%

----------------------------------------------------------------------------

Classification Tag

----------------------------------------------------------------------------

Not significant

----------------------------------------------------------------------------

Thresholds

----------------------------------------------------------------------------

p-value threshold:            0.01

Natural variation:            95

Min control points:           60

Normal values 'males only':   104.700 to 111.7975

Normal values 'females only': 106.800 to 113.500

----------------------------------------------------------------------------

Count Matrices

----------------------------------------------------------------------------

'All' matrix:

       +/+ MDTZ

Low      70    2

Normal 2550   26

High     72    0

'Males only' matrix:

       +/+ MDTZ

Low      36    0

Normal 1252   14

High     34    0

'Females only' matrix:

       +/+ MDTZ

Low      34    2

Normal 1298   12

High     38    0

From the RR method output we can see that the genotype effect for the chloride variable was not classed as significant.

The output of the RR method can be visualized using function *categoricalBarplot* (Figure C2.8).

> categoricalBarplot(resultIMPC_RR)

**Figure C2.8**: Example output of PhenStat *categoricalBarplot* function. Function visualises the categorical data formed from the RR framework as summary percentage data. It reports the percentage of each classification observed for up to three datasets: all data, male only and female only. It is important to note that percentage accuracy is very dependent on the number of readings so it is important to consider the dataset size when interpreting these graphs. Therefore tables showing both the percentage and count values are included in the *summaryOutput*.

The final method you could consider using was the MM. Function’s *testDataset* argument “method” is now set to “MM”.

> resultIMPC_MM<-testDataset(testIMPC,depVariable = "Value", method="MM" )

Warning:

Weight column is not present in dataset.

Equation 'withWeight' can't be used in such a case and has been replaced to 'withoutWeight'.

Information:

Dependent variable: 'Value'.

Information:

Perform all MM framework stages: startModel and finalModel.

Information:

**Method: Mixed Model framework.**

Information:

**Equation: 'withoutWeight'.**

Information:

Calculated values for model effects are: keepBatch=TRUE, keepEqualVariance=TRUE, keepWeight=FALSE, keepSex=TRUE, keepInteraction=FALSE.

> summaryOutput(resultIMPC_MM)

Test for dependent variable:

*** Value ***

Method:

*** Mixed Model framework ***

----------------------------------------------------------------------------

Model Output

----------------------------------------------------------------------------

Genotype effect: 0.1580

Final fitted model: Value ~ Genotype + Sex

Was batch significant? TRUE

Was variance equal? TRUE

Was there evidence of sexual dimorphism? no (p-value 0.3699)

Genotype percentage change Female: 0.4%

Genotype percentage change Male: 0.4%

----------------------------------------------------------------------------

Classification Tag

----------------------------------------------------------------------------

With phenotype threshold value 0.01 - no significant change

----------------------------------------------------------------------------

Model Output Summary

----------------------------------------------------------------------------

                  Value  Std.Error   DF     t-value       p-value

(Intercept)  110.4896631 0.10958683 2599 1008.238517  0.000000e+00

GenotypeMDTZ   0.4376466 0.30948912 2599    1.414094  1.574542e-01

SexMale       -1.9379127 0.05354635 2599  -36.191315 1.288989e-232

From the output we can see that the genotype effect onto chloride variable is not significant according to MM method (*p* value equals to 0.158). Model diagnostic functions allow assisting the quality of the fitted model. Function’s *qqplotGenotype* results are shown in Figure C2.9, *plotResidualsPredicted* results are in Figure C2.10, see Figure C2.11 for the results of the function *qqplotRandomEffects* and Figure C2.12 for the results of the function *qqplotRotatedResiduals*, finally graphic created with the function *boxplotResidualsBatch* is available in Figure C2.13.

> qqplotGenotype(resultIMPC_MM)

**Figure C2.9**: Example output of the PhenStat *qqplotGenotype* function. Data shown is the output from analysis of the chloride measurements from a study on mice comparing wild type strain to knockout strain MDTZ when fitted with the mixed model method. Looking at the example, the residuals for both groups show no systematic deviations from the line indicating the model is fitting this data well.

> plotResidualPredicted(resultIMPC_MM)

**Figure C2.10**: Example output of the PhenStat *plotResidualPredicted* function. This function plots the residuals against the predicted readings for both genotypes. The predicted readings are the values the model would estimate for the variable of interest. Looking at the mice data, we can see that the spread of the residuals is fairly consistent across the range of predicted values.

> qqplotRandomEffects(resultIMPC_MM)

**Figure C2.11**: Example output of the PhenStat *qqplotRandomEffects* function. This function is assessing the assumption that the batch effects are normally distributed. The estimates of the random effects, aka the estimates of the batch effects in this scenario, are called best linear unbiased prediction BLUPs. Here a normal Q-Q plot is used to plot the estimated BLUPs against a normal distribution. So looking at the mice example, the majority of the data points are distributed along the line. There is some systematic deviation at the tails but it is a small percentage of the points and so we can conclude the distribution is not too far from the ideal and the model is a good representation of the data.

> qqplotRotatedResiduals(resultIMPC_MM)

**Figure C2.12**: Example output of the PhenStat *qqplotRotatedResiduals* function. This function, allows the user to consider the normality of the “rotated” and “unrotated” residuals. Looking at the mice example, all of the data points are distributed along the line so we can conclude the distribution is close to the ideal and the model is a good representation of the data.

> boxplotResidualsBatch(resultIMPC_MM)

**Figure C2.13**: Example output of the PhenStat *boxplotResidualsBatch* function. This function allows visualisation to assist the user to assess whether the deviation in the residual is consistent across all the batches and similar in size between the two groups. For the mice example, we can see that the variation in residual is consistent across all the batches and similar in size between the genotype groups.

All three methods (TF, MM and RR) reported a consensus concerning the genotype effect as statistically insignificance for the chloride variable between wild type mice and *Fbxo47* gene knockout mice (MDTZ).
